# Initiation and sustenance of Chinese Wushu practice: a structural equation model validation of the multi-theory model

**DOI:** 10.3389/fpsyg.2026.1815407

**Published:** 2026-05-15

**Authors:** Quan Sun, Liping Ding, Hongyan Zhu, Mei He, Dandan Shen, Yiling Zhu, Dong Zhu

**Affiliations:** 1 School of Wushu, Shanghai University of Sport, Shanghai, China; 2 College of International Studies, Yangzhou University, Yangzhou, China

**Keywords:** Chinese Wushu, health behavior change, initiation, multi-theory model, physical activity, structural equation modeling, sustenance, Wushu practice

## Abstract

**Background:**

Wushu is a culturally embedded and skill-intensive physical activity in China with recognized physical and psychosocial benefits. However, participation remains challenged by difficulties in both initiation and long-term sustenance, and phase-specific behavioral explanations in Wushu contexts are still limited.

**Purpose:**

This study applied the Multi-Theory Model (MTM) of health behavior change to examine factors associated with recalled initiation experiences and current sustenance of Wushu practice among Chinese practitioners.

**Methods:**

A cross-sectional survey was conducted with 349 Wushu practitioners from different provinces and municipalities across China using the Measuring Change in Physical Activity Questionnaire (MCPAQ). Data were analyzed using SPSS 26.0 and AMOS 24.0 through exploratory and confirmatory factor analyses and structural equation modeling to evaluate measurement properties and test hypothesized MTM pathways.

**Results:**

The study provided partial support for MTM's phase distinction, with differential predictors for initiation and sustenance, while highlighting context-specific attenuation of selected constructs. For behavioral initiation, changes in the physical environment (β = 0.436, *p* < 0.001) and behavioral confidence (β = 0.181, *p* = 0.049) were significant predictors, whereas participatory dialogue–advantages (β = 0.159, *p* = 0.067) and participatory dialogue–disadvantages (β = 0.082, *p* = 0.173) were not significant. The initiation model explained 48.3% of the variance in behavioral initiation (*R*^2^ = 0.483). For behavioral sustenance, emotional transformation (β = 0.422, *p* < 0.001) and practice for change (β = 0.302, *p* < 0.001) significantly predicted sustained practice, while changes in the social environment were not significant (β = 0.131, *p* = 0.146). The sustenance model explained 60.7% of the variance in behavioral sustenance (*R*^2^ = 0.607).

**Conclusions:**

These findings provide partial support for the MTM in the context of Wushu practice while highlighting context-specific variability in construct performance within culturally grounded, skill-dependent activities. Structural accessibility appears central to initiation, whereas emotional and self-regulatory processes are key to sustaining practice, informing phase-appropriate strategies for Wushu promotion and health-oriented interventions.

## 1 Introduction

Physical inactivity remains a global public health concern due to its well-established associations with chronic disease, reduced wellbeing, and premature mortality (Bull et al., 2020). As a culturally embedded form of physical activity, Chinese Wushu has demonstrated benefits for cardiovascular fitness, neuromuscular coordination, mental wellbeing, and overall quality of life across diverse populations (Origua Rios et al., 2018; Wang et al., 2025; Xu et al., 2025b). Despite these documented benefits, many individuals encounter difficulties not only in initiating Wushu practice but also in sustaining long-term participation (Clavel et al., 2025; Tu et al., 2025; Wang et al., 2025). Such challenges mirror broader patterns observed in physical activity behavior, where maintaining adherence consistently proves more difficult than initial adoption (Viken et al., 2019; Wang and Hagger, 2023).

Existing research on Wushu has largely focused on its cultural significance, technical pedagogy, physical fitness effects, and psychosocial outcomes (Li and Guo, 2016; Li and Dai, 2021; Chen and Morazuki, 2025). Comparatively little attention has been given to the behavioral mechanisms that shape how individuals begin—and continue—engaging in Wushu practice. In the broader physical activity literature, scholars have increasingly emphasized the importance of distinguishing between behavioral initiation and behavioral sustenance, as these processes are often governed by different psychological and contextual determinants. However, this phase-specific perspective has rarely been applied to Wushu, leaving a gap in understanding how individuals engage with complex, skill-intensive practices such as Wushu over time.

The Multi-Theory Model (MTM) of health behavior change provides a contemporary framework explicitly designed to differentiate initiation from sustenance (Sharma et al., 2021; Kapukotuwa et al., 2024; Islam et al., 2025). By integrating empirically supported constructs drawn from multiple behavioral theories, MTM offers a parsimonious approach to explaining both one-time behavioral adoption and long-term behavioral continuation. While MTM has demonstrated explanatory value across a range of health-related behaviors, its applicability to culturally grounded, skill-based practices such as Wushu has not yet been examined. Investigating whether MTM can account for Wushu participation may therefore advance both theoretical understanding and practical strategies for promoting sustained engagement in traditional physical activities.

Against this background, the present study applies the Multi-Theory Model (MTM) of health behavior change to the context of Wushu practice. By examining Wushu participation through a phase-based behavioral framework, this study aims to make three key contributions. First, it seeks to extend the application of MTM to a culturally embedded and skill-intensive form of physical activity that has received limited attention in health behavior research. Second, it examines the differential psychological and contextual mechanisms underlying the initiation and sustenance of Wushu practice through a structural equation modeling approach. Third, the study is intended to identify theory-informed targets that may guide the development of phase-appropriate strategies for promoting long-term engagement in Wushu. In doing so, this research addresses an important gap in the literature and aligns with broader public health efforts to encourage culturally meaningful and health-enhancing physical activities.

## 2 Theoretical framework and hypotheses development

Chinese Wushu is characterized by demanding motor-skill acquisition, progressive technical development, and participation in culturally and relationally structured learning environments (Cao and Lyu, 2024b). Such activities require individuals to navigate multiple layers of decision-making, involving physical capability, cognitive evaluations, emotional responses, and social expectations (Yang and Huang, 2025). This complexity suggests that Wushu participation cannot be fully understood through models that conceptualize behavior as a single-stage process.

Physical activity behavior research has long been guided by theories such as the Theory of Planned Behavior (TPB), Self-Determination Theory (SDT), and Social Cognitive Theory (SCT), which explain motivation, intention formation, and psychosocial influences (Bandura, 2004; Teixeira et al., 2012; Wang and Hagger, 2023; Xu et al., 2025a). While these models have advanced understanding of exercise behavior, a growing body of evidence highlights their limitations in addressing long-term adherence. Meta-analytic findings repeatedly demonstrate a pronounced “intention–behavior gap,” where strong intentions fail to translate into sustained participation (Rhodes and De Bruijn, 2013; Feil et al., 2023; Kapukotuwa et al., 2024). Further, the psychological processes that drive the adoption of a new behavior often differ from those that sustain it over time, suggesting the importance of a phase-based conceptualization (Rhodes and Sui, 2021). Although sustenance-oriented models exist, their application to complex skill-based practices—such as Wushu—remains limited (Nigg et al., 2008).

The Multi-Theory Model (MTM) provides a structured response to this theoretical need by distinguishing behavior change into initiation and sustenance phases. The initiation phase emphasizes participatory dialogue, behavioral confidence, and changes in the physical environment—reflecting cognitive appraisal, efficacy beliefs, and environmental readiness. The sustenance phase highlights emotional transformation, practice for change, and social-environmental support as mechanisms that enable continued engagement (Nahar et al., 2019; Bashir et al., 2025). Empirical studies across physical activity, nutrition, and preventive health behaviors support MTM's predictive validity, reinforcing the importance of separating early-stage and long-term behavioral determinants (Joveini et al., 2023; Jiang et al., 2024; Huai et al., 2025; Islam et al., 2025).

Wushu's behavioral ecology closely aligns with MTM's dual-phase architecture. To begin training, individuals must evaluate perceived advantages and disadvantages, judge their ability to learn complex skills, and secure access to appropriate training settings—processes matching MTM's initiation constructs (Kim et al., 2015; de Borja et al., 2025; Tu et al., 2025). Sustained Wushu practice requires managing emotional fluctuations, developing self-regulatory routines for consistent training, and relying on interpersonal support from instructors and peers, which correspond to MTM's sustenance constructs (Cao and Lyu, 2024b; Wang et al., 2025). This strong theoretical alignment suggests that MTM is well suited to explaining both short-term and long-term engagement in Wushu.

Despite these conceptual connections, no empirical research has applied the MTM to Wushu. Most existing Wushu scholarship focuses on cultural or pedagogical themes rather than behavioral processes, and phase-based models remain underexplored in this domain. Addressing this gap provides an opportunity to extend MTM to a novel behavioral context and to generate theoretically grounded insights into Wushu participation.

**Figure 1:**
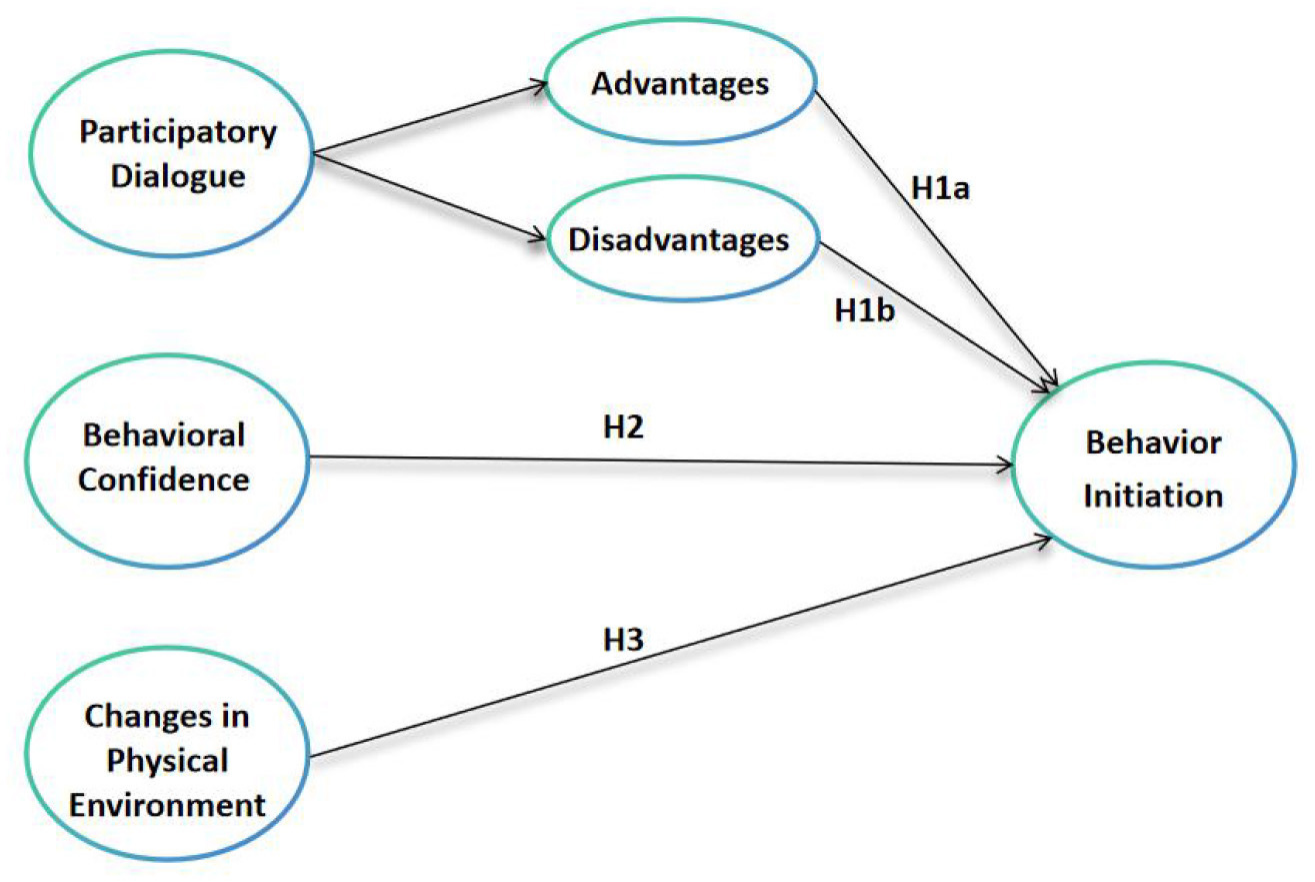
Conceptual framework of the multi-theory model for the initiation of Wushu practice.

**Figure 2:**
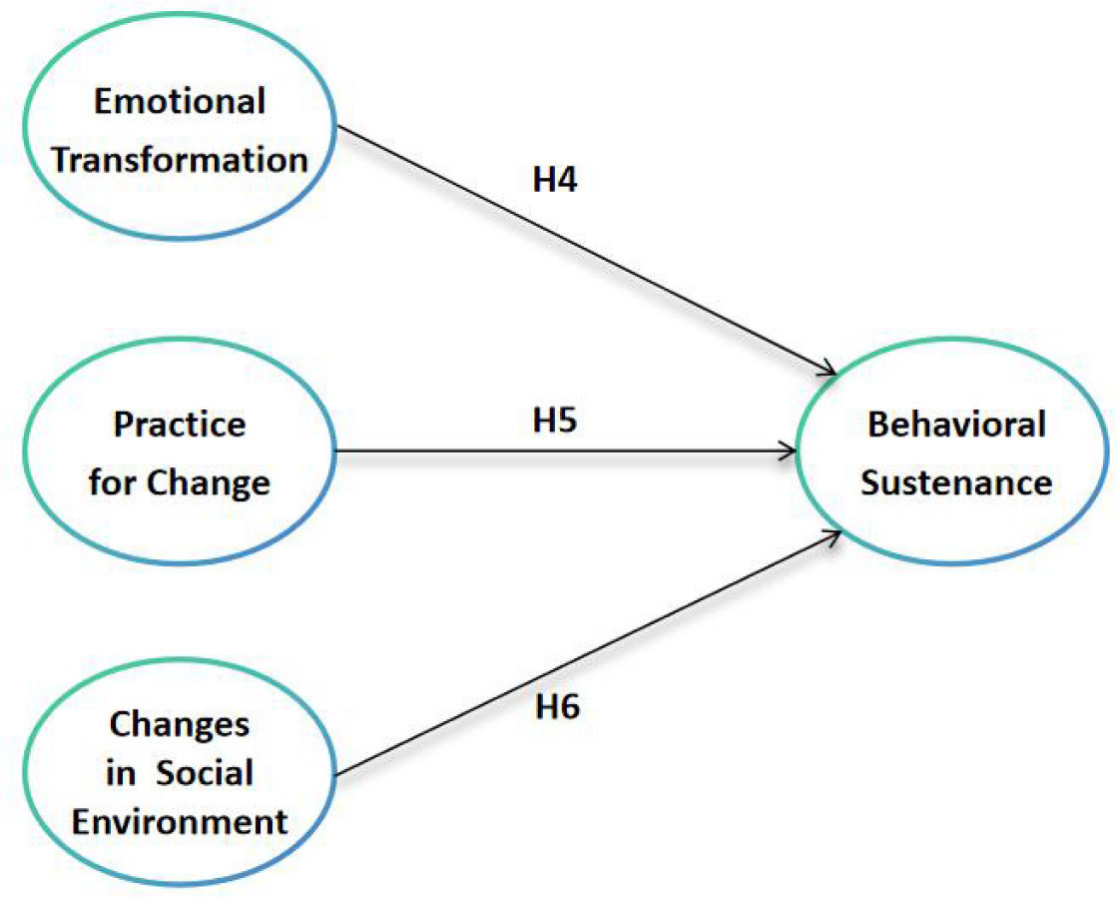
Conceptual framework of the multi-theory model for the sustenance of Wushu practice.

Accordingly, drawing on the Multi-Theory Model and the foregoing theoretical review, the present study proposes a two-phase conceptual framework distinguishing the initiation and sustenance of Wushu practice. The conceptual framework and corresponding research hypotheses are illustrated in Figures 1, 2, which depict the hypothesized relationships among MTM constructs underlying behavioral initiation and behavioral sustenance of Wushu practice, respectively.

Based on the MTM framework and the foregoing review, the following hypotheses are proposed:

**H1a:** participatory dialogue–advantages positively predict behavioral initiation.
**H1b:** lower perceived participatory dialogue–disadvantages positively predict behavioral initiation.
**H2:** behavioral confidence positively predicts behavioral initiation.
**H3:** changes in the physical environment positively predict behavioral initiation.
**H4:** emotional transformation positively predicts behavioral sustenance.
**H5:** practice for change positively predicts behavioral sustenance.
**H6:** changes in the social environment positively predict behavioral sustenance.

## 3 Methods

### 3.1 Study design

This study adopted a cross-sectional survey design to examine behavioral initiation and sustenance of Wushu practice based on the Multi-Theory Model (MTM). The target population consisted of individuals with prior or ongoing experience in Wushu. In this study, “initiation” refers to the early phase of starting regular Wushu practice rather than mere first-time exposure. For initiation-stage measures, respondents were instructed to recall their experiences during the first month when they initially began regular Wushu practice; for sustenance-stage measures, respondents answered based on their current perceived ability to maintain regular Wushu practice over time.

It is important to clarify that the initiation stage assessed in the present study reflects a retrospective evaluation of Wushu practice initiation among individuals with prior Wushu experience, rather than a prospective assessment conducted before behavior onset. All participants had either already initiated or were currently sustaining Wushu practice at the time of data collection. Accordingly, behavioral initiation was operationalized as participants' retrospective recall of their initiation experience, with specific reference to the first month following the commencement of Wushu practice. This retrospective approach has been commonly adopted in health behavior research when recruiting individuals without prior behavioral experience is impractical, and allows for the examination of perceived psychological and environmental conditions associated with successful initiation (Prince et al., 2008; Rhodes and De Bruijn, 2013). Therefore, the initiation-related constructs in the present study capture participants' perceived capability and contextual conditions during their initiation phase, rather than their pre-behavioral intentions or decision-making processes prior to engagement.

In addition, in the present study, Wushu was operationally defined as a broad category encompassing diverse forms of Chinese martial arts practice. This included traditional styles, contemporary competitive Wushu, as well as various routines and combat-oriented forms involving both empty-hand techniques (e.g., fist styles) and weapon-based practices (e.g., sword, saber, spear, and staff). Participants were eligible for inclusion if they engaged in any form of Chinese Wushu practice, regardless of specific style or sub-discipline.

### 3.2 Participants and sampling

A total of 400 questionnaires were distributed, of which 378 were returned (response rate = 94.5%). After data screening, 29 responses were excluded due to insufficient variance, unrealistically short completion times, or incomplete responses. The final analytical sample consisted of 349 Wushu practitioners with valid and usable data.

Participants were recruited using purposive sampling combined with snowball sampling, targeting individuals with documented experience in Chinese Wushu practice. Inclusion criteria required: (a) prior engagement in at least one form of Chinese Wushu, and (b) the ability to independently understand and complete the questionnaire. Participants were excluded if they had never practiced Chinese Wushu, were unable to complete the questionnaire independently, or provided invalid responses (e.g., extremely short completion times, straight-line patterns, or incomplete or contradictory answers).

To enhance ecological validity, participants were recruited from multiple Wushu-related settings—including Wushu schools, university training centers, community groups, competition venues, and online networks—located in multiple provinces and municipalities across China. This recruitment strategy resulted in a heterogeneous sample with variation in age, training experience, learning mode (self-directed, coach–student, or master–disciple), and competitive exposure. Such diversity was intentionally retained to test the robustness of MTM constructs within real-world Wushu participation contexts.

Sample size adequacy was evaluated using established criteria for structural equation modeling (SEM). According to established guidelines for structural equation modeling, a minimum of 10–20 participants per latent variable is recommended, with an absolute minimum sample size of 200 cases for stable model estimation. With nine latent constructs in the MTM, the recommended sample size can be expressed as: *N* ≥ 10 × k, where k represents the number of latent variables (Byrne, 2016). Based on this criterion, a minimum sample size of 90–180 participants was required, which was exceeded by the final sample of 349.

In addition to these SEM-specific guidelines, a supplementary power analysis was conducted using G^*^Power to provide an approximate and conservative check of statistical power for detecting medium-sized effects in key predictive paths, modeled as multiple regression relationships. Following Cohen's criteria (*f*^2^ = 0.15, α = 0.05, power = 0.80) (Faul et al., 2007; Cohen, 2013), the analysis indicated that a minimum sample size of 146 participants would be sufficient. This supplementary analysis was intended to support the adequacy of the sample size rather than to serve as a formal power estimation for SEM. Taken together, these considerations indicate that the achieved sample size was sufficient for the planned EFA, CFA, and SEM analyses.

### 3.3 Instrument

A measurement instrument based on the Multi-Theory Model (MTM) was employed in this study, using the Measuring Change in Physical Activity Questionnaire (MCPAQ), which was originally developed within the MTM theoretical framework. The instrument was used to assess key psychological and environmental factors associated with Chinese Wushu practice across two behavioral phases: initiation and sustenance. Formal written permission to use the MCPAQ was obtained from the original author, Manoj Sharma. To improve contextual relevance while preserving construct meaning, the original MCPAQ items were semantically adapted to the Wushu context by the research team. The adapted wording was further reviewed by teachers, academic peers, and individuals familiar with Wushu practice to ensure linguistic clarity, contextual appropriateness, and consistency with the intended construct meanings. Based on this review, minor wording refinements were made before formal data collection.

In the present study, the original MTM constructs and their theoretical definitions were fully retained. No constructs were added, removed, merged, or redefined. All measurement adaptations were confined to the item level and were implemented solely to enhance contextual relevance to Chinese Wushu practice. Specifically, all items from the original questionnaire were retained and semantically contextualized to reflect the practice characteristics, training settings, and experiential features of Wushu, without altering the underlying construct meanings. The MTM-based instrument comprised nine latent constructs corresponding to the initiation and sustenance models. All items were rated on a 7-point Likert scale ranging from 1 (strongly disagree) to 7 (strongly agree), with higher scores indicating more favorable psychological, behavioral, or environmental conditions for Wushu practice. Items assessing participatory dialogue–disadvantages were negatively worded and were reverse-coded prior to statistical analysis, so that higher scores reflect fewer perceived disadvantages of Wushu practice.

#### 3.3.1 Initiation model

According to the MTM framework, the initiation model included four antecedent constructs—participatory dialogue–advantages (PDadv), participatory dialogue–disadvantages (PDdis), behavioral confidence (BC), and changes in the physical environment (CPE)—as well as behavioral initiation (BI) as the outcome construct. In this study, all constructs within the initiation model demonstrated acceptable internal consistency reliability. Specifically, Cronbach's α coefficients were 0.776 for PDadv, 0.799 for PDdis, 0.867 for BC, 0.737 for CPE, and 0.838 for BI, all exceeding the recommended threshold of 0.70, indicating satisfactory reliability of the initiation model measures.

#### 3.3.2 Sustenance model

Based on MTM's conceptualization of behavioral sustenance, the sustenance model comprised three antecedent constructs—emotional transformation (ET), practice for change (PC), and changes in the social environment (CSE)—and behavioral sustenance (BS) as the outcome construct. Results indicated that all latent variables in the sustenance model exhibited acceptable internal consistency reliability. Cronbach's α coefficients were 0.773 for ET, 0.754 for CSE, 0.762 for PC, and 0.878 for BS, suggesting that the measurement instrument demonstrated good reliability in the present sample.

### 3.4 Ethical approval

Ethical approval for this study was obtained from the Institutional Review Board of Shanghai University of Sport (Approval No.: 102772025RT306). All procedures were conducted in accordance with the Declaration of Helsinki. Prior to participation, all participants were informed about the purpose and voluntary nature of the study. Written informed consent was obtained from all adult participants, and for participants aged 12–17 years, written parental or legal guardian consent was obtained in addition to participant assent. Participation was entirely voluntary, and participants could withdraw from the study at any time without any negative consequences. All data were collected anonymously, with no personally identifiable information recorded. The data were used solely for academic research purposes and were reported only in aggregated form to ensure participant confidentiality.

### 3.5 Procedure

Questionnaires were distributed at Wushu schools, university training centers, community Wushu groups, competition venues, and online platforms. Participation was voluntary and anonymous. Respondents typically completed the questionnaire in 10–20 minutes. To reduce potential common method bias, anonymity and confidentiality were emphasized, no right or wrong answers were indicated, and participants were encouraged to respond honestly. To ensure data quality, multiple checks were applied, including attention to inconsistent responses, duplicated patterns, extremely short completion times, missing data, and near-zero variance in answers. Invalid cases were removed prior to analysis. Analyses were conducted using SPSS 26.0 and AMOS 24.0. Data screening included checks for missing values, univariate and multivariate outliers (*Z*-scores and Mahalanobis distance), normality (skewness and kurtosis), and multicollinearity diagnostics. Common method bias was examined using Harman's single-factor test. Descriptive statistics were computed for all latent variables. A three-stage analytic strategy was used to evaluate the psychometric and structural properties of the MTM:

(1) Exploratory factor analysis (EFA): given that the MCPAQ is a theoretically grounded and previously validated instrument, exploratory factor analysis (EFA) was conducted to examine the dimensionality and item performance of the semantically adapted scale in a new Chinese Wushu context. The purpose of the EFA was to screen items and refine indicator retention, rather than to develop a new instrument. Principal axis factoring with Promax rotation was used to identify items that performed inadequately in this specific cultural and behavioral setting.
(2) Confirmatory factor analysis (CFA): evaluation of measurement model fit, factor loadings, and construct validity (CR, AVE, HTMT).
(3) Structural equation modeling (SEM): separate initiation and sustenance models were tested using maximum likelihood estimation. Model fit, standardized path coefficients, and explained variance (*R*^2^) were reported to evaluate the hypothesized MTM relationships.

**Figure 3:**
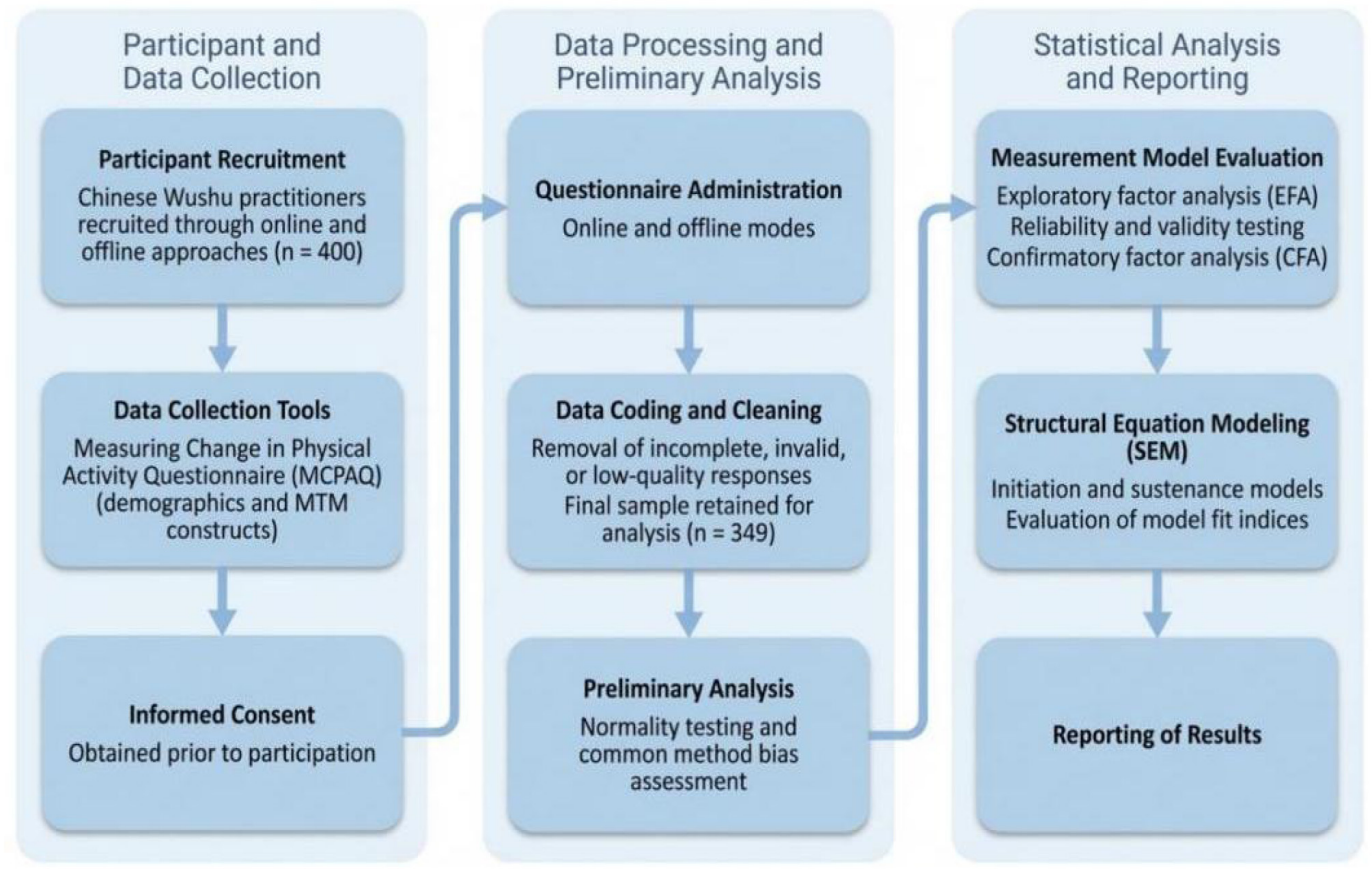
Flow diagram of research.

This cross-sectional study was reported in accordance with the STROBE guidelines. The procedures for participant recruitment, data collection, data screening, and statistical analyses are illustrated in Figure 3.

## 4 Results

### 4.1 Preliminary analyses

Prior to conducting EFA, CFA, and SEM, a series of preliminary analyses were performed to evaluate data quality, assess statistical assumptions, and ensure suitability for multivariate modeling. No missing data were detected, and all 349 cases were retained for screening.

Univariate normality was assessed using skewness and kurtosis. Across the 35 initial measurement items, skewness values ranged from −1.12 to 0.88 and kurtosis from −0.95 to 2.31, all within the recommended thresholds of |skewness| < 3 and |kurtosis| < 10 (Kline, 2016), indicating acceptable univariate distributions. Multivariate normality, however, was not supported. Mardia's multivariate kurtosis was elevated (*z* = 28.47, *p* < 0.001), suggesting deviation from multivariate normality. Given this violation, the Bollen–Stine bootstrap procedure was employed to assess the robustness of the chi-square model fit under non-normal conditions. The bootstrap-corrected fit indices were highly consistent with those obtained from the maximum likelihood (ML) estimation, indicating that overall model fit conclusions were stable. Accordingly, all parameter estimates, path coefficients, and hypothesis tests reported in this study are based on ML estimation.

Multivariate outliers were examined using Mahalanobis distance (*D*^2^). A total of 31 cases exceeded the critical χ^2^ value at *p* < 0.001. Inspection of individual response patterns showed no careless responding, straight-lining, or semantic contradictions; thus, consistent with recommendations by SEM reporting guidance, these cases were retained (Byrne, 2016; Hair and Alamer, 2022), as statistical outlier flags alone do not justify exclusion without conceptual evidence of invalidity.

To assess multicollinearity, variance inflation factors (VIF) and tolerance values were computed for all indicators. VIF values ranged from 1.22 to 2.48, and all tolerance values exceeded 0.40, well below the thresholds indicating problematic multicollinearity (VIF > 5). These results suggested that redundancy among predictors was not a concern.

To evaluate common method bias (CMB), Harman's single-factor test was performed using all 35 initial items. The first unrotated factor accounted for 34.43% of the total variance—substantially below the 50% threshold (Podsakoff et al., 2003)—indicating that no single factor dominated the covariance structure. A single-factor confirmatory factor analysis model also demonstrated poor model fit relative to the hypothesized MTM measurement model, further suggesting that CMB was unlikely to significantly bias the findings.

Collectively, these screening results supported the adequacy of the dataset and justified proceeding with EFA, CFA, and SEM.

### 4.2 Demographic characteristics

A total of 349 valid responses were included in the analyses. The sample consisted of 73.9% males and 26.1% females. Participants ranged widely in age, with 35.5% aged 12–17, 47.0% aged 18–44, 13.5% aged 45–59, and 4.0% aged 60 or above. Educational levels were diverse, with 42.1% having completed junior–senior high or vocational education and 30.4% holding a college or undergraduate degree. Wushu experience ranged from 1 to 52 years, with most respondents reporting 3–10 years of practice. Learning modes were primarily coach–student instruction (67.0%), followed by master–disciple transmission (24.9%). Nearly half (43.0%) reported having family members who also practiced Wushu. An overview of demographic characteristics is presented in Table 1. Notably, the broad range of training experience (1–52 years) and the presence of both recreational and competition-involved practitioners indicate that participants may occupy different points along the initiation–sustenance continuum. Accordingly, the present analyses focus on testing MTM's phase-specific pathways at the model level, while acknowledging potential heterogeneity in subgroup mechanisms.

**Table 1:** Demographic characteristics of the participants (*N* = 349).

Variable	Category	*n*	%
Gender	Male	258	73.9
	Female	91	26.1
Age group	12–17 years	124	35.5
	18–44 years	164	47.0
	45–59 years	47	13.5
	≥60 years	14	4.0
Education level	Primary school	38	10.9
	Junior–senior high school/vocational	147	42.1
	Junior college/undergraduate	106	30.4
	Master's or doctoral degree	58	16.6
Years of Wushu experience	1–3 years	78	22.3
	4–10 years	191	54.7
	≥11 years	80	22.9
Learning mode	Self-directed learning	28	8.0
	Coach–student system	234	67.0
	Master–disciple tradition	87	24.9
Family members practicing Wushu	Yes	150	43.0
	No	199	57.0
Competition experience	None	43	12.3
	Occasionally	185	53.0
	Frequently	121	34.7

### 4.3 Exploratory factor analysis

EFA was conducted prior to CFA to evaluate the dimensionality and psychometric performance of the contextualized items. The original measurement pool consisted of 35 items. The Kaiser–Meyer–Olkin (KMO) measure of sampling adequacy reached 0.922, indicating excellent suitability for factor analysis, and Bartlett's test of sphericity was significant, χ^2^(595) = 6777.954, *p* < 0.001, supporting the factorability of the correlation matrix.

#### 4.3.1 First-round extraction (unconstrained)

Using principal axis factoring with eigenvalues greater than 1, the initial solution produced a multi-factor structure. The first factor accounted for 34.43% of the variance, with a clear inflection observed in the scree plot after the fourth factor, suggesting a naturally emerging four- to five-factor structure. Although informative, this pattern did not align with the theoretically expected nine-factor MTM structure, supporting the need for a theory-driven extraction.

#### 4.3.2 Second-round extraction (fixed nine-factor solution)

A fixed nine-factor solution was subsequently specified to evaluate the extent to which the empirical structure corresponded to the MTM's theoretical constructs. Pattern loadings generally supported the hypothesized structure; however, several items demonstrated inadequate psychometric properties. Specifically, four items exhibited poor communalities (< 0.40), including PD1 (0.348), PD9R (0.206), CPE3 (0.265), and ET3 (0.287). Additional items (e.g., BS3, PD10, PC1, CSE3) demonstrated weak primary loadings (< 0.50), while PC3 and PD1 showed overall suppressed loadings across all factors, indicating problematic construct alignment. One item (BI2) displayed minimal discrimination between two factors (Δloading = 0.029), suggesting cross-loading concerns.

#### 4.3.3 Final EFA results and transition to CFA

Four items—PD1, PD9R, CPE3, and PC3—were removed based on their consistently low communalities, suppressed primary loadings, or conceptually inconsistent loading patterns across iterations. After their removal, the factor structure was re-estimated with 31 items, producing a clearer and more stable nine-factor configuration. Communalities improved (range = 0.33–0.81), all primary loadings exceeded 0.50, and the resulting structure demonstrated satisfactory interpretability consistent with MTM's theoretical domains. Because some borderline items (e.g., BI2, ET3, BS3, CSE3, PD10, PC1) improved after refinement and maintained theoretical coverage, they were retained for evaluation in the confirmatory factor analysis.

Thus, the final EFA solution retained 31 items across the nine theorized MTM factors and was carried forward to the CFA stage. During CFA, one additional item (PD3) was subsequently removed due to its relatively low standardized loading and its suppressing effect on the AVE of the PDadv construct, resulting in the final 30-item measurement model used in all subsequent analyses.

### 4.4 Confirmatory factor analysis

A confirmatory factor analysis (CFA) was then conducted on the 31-item, nine-factor structure retained from the EFA to further evaluate the measurement properties of the MTM model among Chinese Wushu practitioners. Based on the initial CFA results, one item from Participatory Dialogue–Advantages (PD3) showed a relatively low standardized loading (0.48) and suppressed the AVE of the PDadv construct. Given that PDadv still retained three strong indicators and that model parsimony is recommended when an item contributes only marginally to construct reliability (Härdle et al., 2024), PD3 was removed. The final CFA was therefore performed using the remaining 30 items, and the resulting measurement model demonstrated substantially improved psychometric quality.

#### 4.4.1 Model fit

The revised nine-factor measurement model achieved satisfactory fit: χ^2^/df = 2.73, CFI = 0.930, TLI = 0.917, RMSEA = 0.070 (90% CI = 0.065–0.075), and SRMR = 0.059. All indicators met commonly accepted SEM thresholds (CFI/TLI ≥ 0.90; RMSEA ≤ 0.08; SRMR ≤ 0.08), indicating good global model fit (Hu and Bentler, 1999).

#### 4.4.2 Factor loadings and convergent validity

All standardized factor loadings ranged from 0.637 to 0.954, exceeding the recommended threshold of 0.50, with all paths significant at *p* < 0.001, indicating strong item–factor relationships. Composite reliability (CR) values ranged from 0.747 to 0.902, all surpassing the 0.70 benchmark. Average variance extracted (AVE) values ranged from 0.498 to 0.757. Although the AVE for CPE was slightly below the conventional threshold (AVE = 0.498), the construct was retained due to its theoretical relevance and adequate composite reliability (CR = 0.747). Values close to 0.50 are commonly considered acceptable in applied measurement models when reliability evidence is sufficient (Fornell and Larcker, 1981a,b). All CR and AVE results are presented in Table 2.

**Table 2:** Composite reliability (CR), average variance extracted (AVE), and Cronbach's α.

Construct	CR	AVE	Cronbach's α
PDadv	0.779	0.541	0.776
PDdis	0.800	0.501	0.799
BC	0.871	0.578	0.867
CPE	0.747	0.498	0.737
BI	0.868	0.690	0.838
ET	0.813	0.603	0.773
CSE	0.760	0.514	0.754
PC	0.783	0.553	0.762
BS	0.902	0.757	0.878

PDadv, participatory dialogue–advantages; PDdis, participatory dialogue–disadvantages (reverse-coded; higher scores indicate fewer perceived disadvantages); BC, behavioral confidence; CPE, changes in physical environment; BI, behavioral initiation; ET, emotional transformation; PC, practice for change; CSE, changes in social environment; BS, behavioral sustenance.

#### 4.4.3 Discriminant validity

Discriminant validity was evaluated using the Fornell–Larcker criterion. For each construct, the square root of AVE exceeded all corresponding inter-factor correlations (Table 3), indicating clear discriminant boundaries among the nine MTM constructs.

**Table 3:** Fornell–Larcker criterion and latent factor correlations.

Construct	PDadv	PDdis	BC	CPE	BI	ET	CSE	PC	BS
PDadv	**0.735**								
PDdis	0.441^***^	**0.708**							
BC	0.681^***^	0.315^***^	**0.760**						
CPE	0.383^***^	0.305^***^	0.595^***^	**0.705**					
BI	0.487^***^	0.346^***^	0.583^***^	0.625^***^	**0.830**				
ET	0.564^***^	0.350^***^	0.576^***^	0.500^***^	0.779^***^	**0.776**			
CSE	0.475^***^	0.234^**^	0.616^***^	0.615^***^	0.702^***^	0.769^***^	**0.717**		
PC	0.541^***^	0.277^***^	0.616^***^	0.465^***^	0.628^***^	0.702^***^	0.686^***^	**0.744**	
BS	0.460^***^	0.354^***^	0.612^***^	0.595^***^	0.814^***^	0.740^***^	0.669^***^	0.694^***^	**0.870**

Diagonal values are √AVE.

Off-diagonal values are latent correlations.

Discriminant validity is supported when √AVE > correlations.

^***^p < 0.001; ^**^p < 0.01.

Bold values indicate the square roots of AVE on the diagonal.

Additionally, HTMT ratios ranged from 0.234 to 0.890 (Table 4), remaining below the recommended 0.90 threshold for conceptually related constructs (Henseler et al., 2015), further supporting discriminant validity.

**Table 4:** HTMT matrix for discriminant validity.

Construct	PDadv	PDdis	BC	CPE	BI	ET	CSE	PC	BS
PDadv	—								
PDdis	0.449	—							
BC	0.678	0.314	—						
CPE	0.378	0.294	0.596	—					
BI	0.486	0.357	0.598	0.640	—				
ET	0.561	0.356	0.571	0.544	0.806	—			
CSE	0.474	0.237	0.619	0.615	0.710	0.779	—		
PC	0.500	0.234	0.590	0.469	0.665	0.707	0.699	—	
BS	0.460	0.356	0.625	0.606	**0.890**	0.749	0.679	0.730	—

#### 4.4.4 Final measurement model

**Figure 4:**
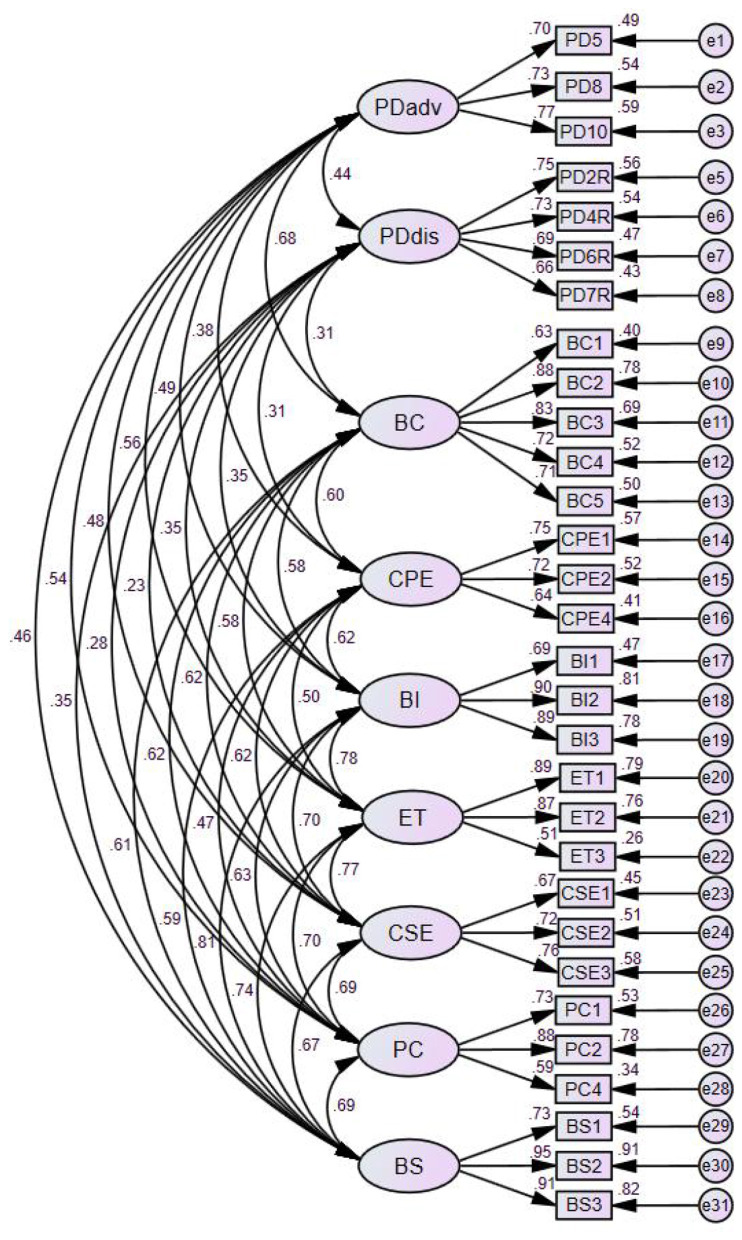
Final confirmatory factor analysis (CFA) measurement model.

The final 30-item MTM measurement structure demonstrated sound reliability, convergent validity, and discriminant validity. The standardized factor loadings and latent variable structure are shown in Figure 4.

Descriptive statistics for the nine MTM latent variables based on the final 30-item measurement model are presented in Table 5. Mean scores across the constructs ranged from moderate to high, indicating generally positive perceptions of behavioral confidence, environmental support, emotional regulation, and sustained engagement in Wushu practice. Standard deviations ranged from 0.88 to 1.22, indicating adequate variability across participants. In addition, construct-level skewness (−1.20 to 0.20) and kurtosis (−0.54 to 1.29) were both within acceptable ranges, further supporting the distributional suitability of the retained indicators for subsequent structural modeling.

**Table 5:** Descriptive statistics for the nine MTM latent variables in the final measurement model.

Construct	Mean	SD	Skewness	Kurtosis
PDadv	5.78	1.01	−0.71	0.31
PDdis	4.55	1.36	0.10	−0.64
BC	5.63	1.04	−0.56	−0.27
CPE	6.04	0.98	−1.14	1.09
BI	6.04	1.03	−1.07	0.30
ET	6.08	0.88	−1.07	1.29
CSE	5.66	1.05	−0.47	−0.30
PC	6.13	0.90	−0.90	0.06
BS	6.09	1.06	−1.20	0.65

### 4.5 Structural equation modeling results

#### 4.5.1 Overall model fit

Structural equation modeling (SEM) was conducted to examine the initiation and sustenance mechanisms proposed by the Multi-Theory Model (MTM) in the context of Wushu practice. Both models demonstrated satisfactory global fit according to conventional criteria (Hu and Bentler, 1999).

The initiation model (PDadv, PDdis, BC, and CPE predicting BI) exhibited good fit: χ^2^ (125) = 225.396, χ^2^/df = 1.803, GFI = 0.933, AGFI = 0.908; CFI = 0.965, TLI = 0.957, RMSEA = 0.048 (90% CI = 0.038–0.058). All indices met or exceeded recommended thresholds (CFI/TLI ≥ 0.95; RMSEA ≤ 0.06), indicating strong overall fit.

The sustenance model (CSE, ET, and PC predicting BS) also demonstrated acceptable fit: χ^2^(48) = 125.021, χ^2^/df = 2.605, GFI = 0.945, AGFI = 0.911; TLI = 0.955, CFI = 0.968, RMSEA = 0.068 (90% CI = 0.053–0.083).

Overall, both SEM models met or exceeded recommended thresholds for good model fit, providing a robust basis for interpretation of structural paths.

#### 4.5.2 Structural path estimates

**Figure 5:**
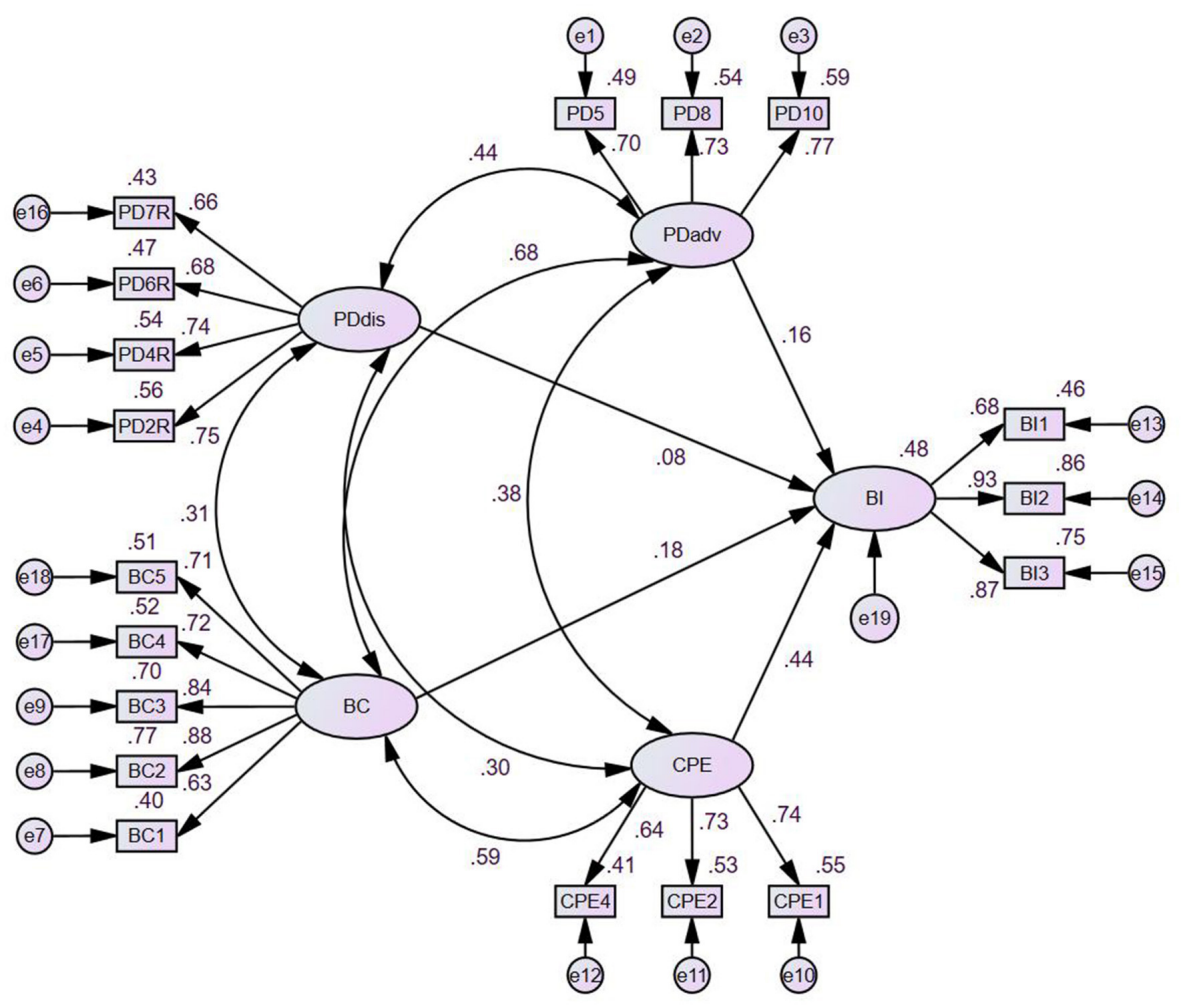
Structural model for behavioral initiation of Wushu.

Standardized path coefficients for the initiation model are reported in Table 6 and depicted in Figure 5. Among the four predictors, changes in the physical environment (CPE) emerged as the strongest determinant of Wushu initiation (β = 0.436, *p* < 0.001), supporting H3. Behavioral confidence (BC) also showed a small but significant effect on initiation (β = 0.181, *p* = 0.049), supporting H2. In contrast, neither participatory dialogue–advantages (PDadv) nor participatory dialogue–disadvantages (PDdis) significantly predicted initiation of Wushu practice (β = 0.159, *p* = 0.067; β = 0.082, *p* = 0.173, respectively), providing no support for H1a or H1b. Collectively, the four MTM constructs explained 48.3% of the variance in initiation behavior (*R*^2^ = 0.483), indicating substantial explanatory power for a first-time application in a new cultural–behavioral context.

**Table 6:** Standardized path coefficients for initiation and sustenance models.

Model/path	β	*p*-value	Supported	*R^2^*
Initiation model				
PDadv → BI (H1a)	0.159	0.067	No (H1a not supported)	**0.483 (BI)**
PDdis → BI (H1b)	0.082	0.173	No (H1b not supported)	
BC → BI (H2)	0.181	0.049	**Yes (H2 supported)**	
CPE → BI (H3)	0.436	< 0.001	**Yes (H3 supported)**	
Sustenance model				
ET → BS (H4)	0.422	< 0.001	**Yes (H4 supported)**	**0.607 (BS)**
PC → BS (H5)	0.302	< 0.001	**Yes (H5 supported)**	
CSE → BS (H6)	0.131	0.146	No (H6 not supported)	

Standardized coefficients are reported.

PDdis is reverse-coded (higher scores indicate fewer perceived disadvantages).

Bold values indicate the R^2^ values of the endogenous constructs (BI and BS).

**Figure 6:**
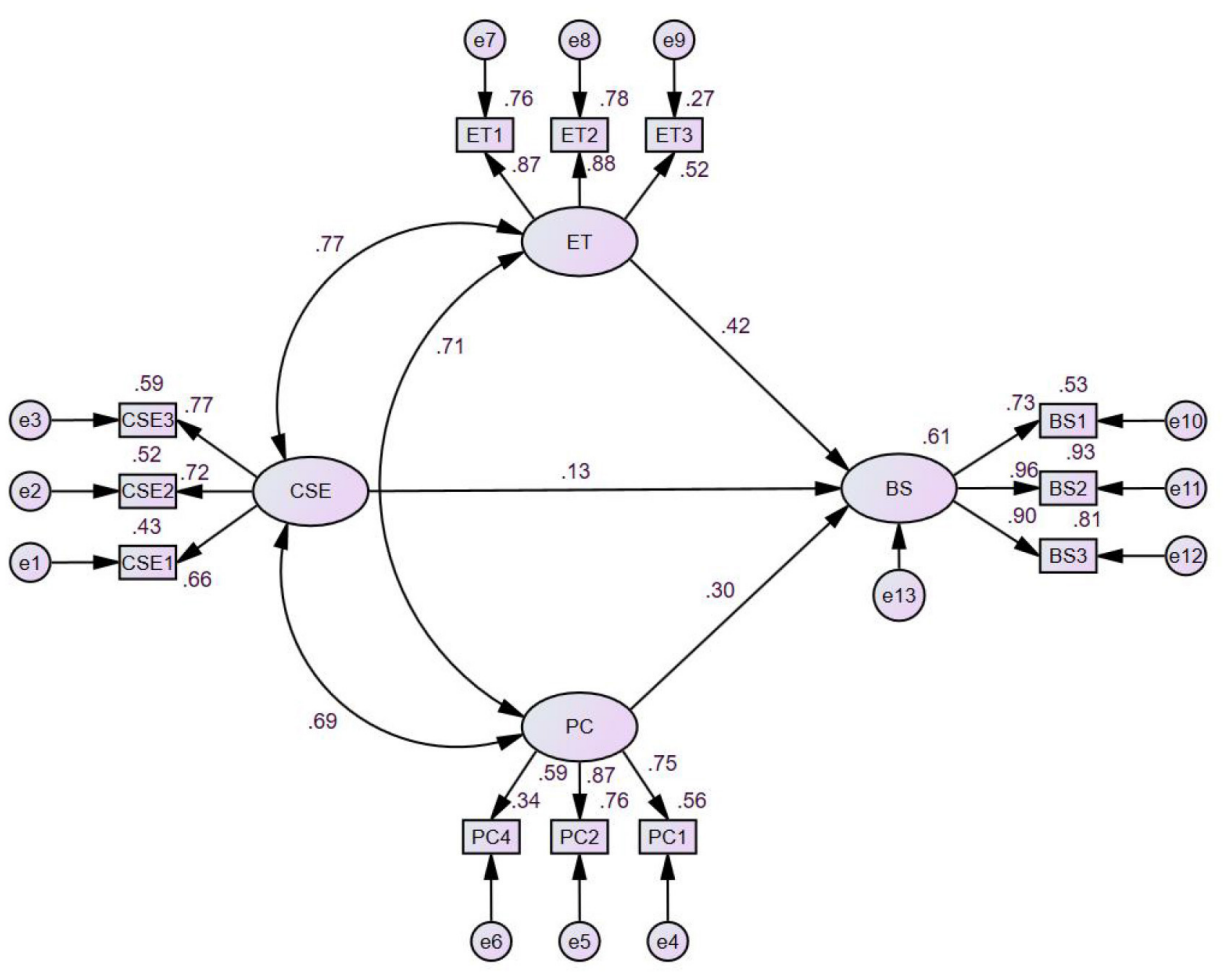
Structural model for behavioral sustenance of Wushu.

Standardized path coefficients for the sustenance model are likewise reported in Table 6 and illustrated in Figure 6. For behavioral sustenance, both emotional transformation (ET) and practice for change (PC) exerted significant positive effects on sustaining Wushu practice (β = 0.422, *p* < 0.001; β = 0.302, *p* < 0.001), supporting H4 and H5. However, changes in the social environment (CSE) did not significantly predict sustenance (β = 0.131, *p* = 0.146), and thus H6 was not supported. The three MTM constructs jointly accounted for 60.7% of the variance in behavioral sustenance (*R*^2^ = 0.607), reflecting strong predictive capacity for continued engagement.

## 5 Discussion

The present study applied the Multi-Theory Model (MTM) to investigate phase-specific behavioral mechanisms related to recalled initiation experiences and current sustenance of Wushu practice, addressing a notable gap in theoretical research on Wushu engagement. Structural equation modeling results partially supported MTM's two-phase structure. In relation to recalled initiation experiences, changes in the physical environment emerged as the most salient associated factor, followed by a smaller yet significant contribution of behavioral confidence, whereas neither dimension of participatory dialogue demonstrated a meaningful effect. For current sustenance, emotional transformation and practice for change significantly predicted continued engagement, whereas changes in the social environment were not influential. Taken together, these findings provide partial support for the MTM in the Wushu context while also revealing context-specific variation in the relative importance of its constructs, offering a basis for deeper interpretation of behavioral, psychological, and cultural processes.

Initiation of Wushu practice was primarily shaped by structural and efficacy-related determinants. The strong predictive power of environmental readiness (β = 0.436, *p* < 0.001) underscores the importance of accessible training spaces, qualified instructors, and institutional opportunities in enabling beginners to embark on Wushu practice. This is consistent with broader physical activity literature demonstrating that built environment factors substantially influence exercise uptake (Smith et al., 2017; An et al., 2019; Sallis et al., 2020). MTM-based studies similarly highlight the central role of environmental facilitation in health behavior initiation (Bashir et al., 2025; Islam et al., 2025). The prominence of environmental determinants in the Wushu context likely reflects the technical and cultural specificity of Wushu training, which requires structured settings and expert guidance rather than casual or improvised participation.

Behavioral confidence also contributed significantly to initiation (β = 0.181, *p* = 0.049), aligning with MTM's emphasis on self-efficacy-like constructs (Nahar et al., 2019; Bashir et al., 2025). However, its weaker effect relative to environmental factors may indicate that confidence is secondary to access-related conditions when engaging in skill-intensive practices. This interpretation resonates with research in skill-dependent sports showing that early self-efficacy development is often contingent on structured exposure and supportive learning environments (Warner et al., 2014). Given that the sample largely comprised individuals already interested in or exposed to Wushu, variability in behavioral confidence may have been insufficient to exert stronger predictive influence.

Contrary to MTM expectations, neither perceived advantages nor perceived disadvantages significantly predicted behavioral initiation in the present sample (PDadv predicted BI: β = 0.159, *p* = 0.067; PDdis predicted BI: β = 0.082, *p* = 0.173). This pattern can be partially explained by the characteristics of the study population, which consisted exclusively of current or former Wushu practitioners, indicating that an initial evaluative decision regarding participation had already been made prior to survey completion. This pattern is consistent with prior MTM applications in which target populations already held favorable orientations toward the behavior, thereby attenuating the explanatory power of evaluative constructs at initiation (Sharma et al., 2022). In this sense, the non-significance of participatory dialogue may be more appropriately interpreted as a sampling-related attenuation of evaluative variance than as a theoretical limitation of the MTM itself. Consistent with this interpretation, descriptive statistics revealed relatively high perceived advantages (M = 5.84, SD = 0.91) and moderate scores on PDdis (reverse-coded; M = 4.29, SD = 1.22), accompanied by limited dispersion and moderate skewness (PDadv skewness = −0.72; PDdis skewness = 0.20), suggesting a restricted range of evaluative perceptions during the initiation phase and reduced statistical sensitivity. Beyond these distributional effects, initiation in the context of Wushu may be shaped less by explicit cost–benefit deliberation and more by affective and identity-related processes, such as intrinsic motivation, cultural imagination, role modeling, and early exposure through family, school, or organized training environments. For culturally embedded and skill-intensive practices, the decision to initiate regular participation may precede, or even bypass, conscious evaluations of advantages and disadvantages (Stevens et al., 2017; Murray et al., 2022). Overall, these findings do not challenge the MTM framework itself; rather, they suggest that under retrospective assessment conditions and within a sample composed exclusively of individuals already exposed to or engaged in Wushu, the explanatory role of evaluative constructs may be attenuated. In such culturally embedded and institutionally scaffolded activities, access- and efficacy-related factors—such as behavioral confidence (β = 0.181, *p* = 0.049) and changes in the physical environment (β = 0.436, *p* < 0.001)—may therefore play a more prominent role in recalled initiation experiences. This pattern further suggests that, in culturally embedded and institutionally scaffolded activities such as Wushu, initiation-related perceptions may be shaped less by deliberative cost–benefit appraisal than by exposure, identity formation, and structural opportunity.

In contrast to initiation, the sustenance of Wushu practice was most strongly driven by internal self-regulatory mechanisms. Emotional transformation demonstrated a substantial effect on continued engagement, emphasizing the importance of practitioners' ability to manage frustration, reinterpret difficulty, and channel emotional fluctuations constructively. This aligns with research identifying affective self-regulation as a key determinant of long-term adherence in complex physical activities (Potoczny et al., 2022; Herzog-Krzywoszanska et al., 2024). Wushu traditions explicitly emphasize discipline, perseverance, and emotional cultivation, which may heighten the relevance of MTM's emotional transformation construct (Tu et al., 2025; Xu et al., 2025b).

Practice for change also significantly predicted sustenance, reinforcing the role of self-regulatory planning, goal setting, and routine formation in forming stable, long-term practice habits. Evidence from physical activity research consistently shows that structured self-regulation strategies substantially improve adherence (Kaushal and Rhodes, 2015). In Wushu, where individual practice between formal sessions is essential for technical progression, the ability to maintain structured self-practice routines becomes a particularly decisive factor in sustaining long-term engagement. This finding aligns with qualitative studies attributing persistence in Wushu to disciplined self-practice, structured repetition, and self-monitoring (Cao and Lyu, 2024a,b; Wang et al., 2025). Together, emotional transformation and practice for change accounted for a large proportion of variance in behavioral sustenance (*R*^2^ = 0.607), underscoring the weight of internal regulatory processes in long-term engagement.

Contrary to H6, changes in the social environment (CSE) did not significantly predict Wushu sustenance (β = 0.131, *p* = 0.146), diverging from previous MTM applications in other domains. It is important to note that this non-significance is unlikely due to a lack of variance in the data, as the standard deviation for CSE (SD = 0.90) was comparable to that of significant predictors like Emotional Transformation (SD = 0.88; see Table 5). Instead, this finding may reflect the unique “saturation effect” of social support within the Wushu ecosystem. Unlike general physical activities where social support is often variable and transactional, Wushu is embedded in a rigid Master-Disciple (Shifu-Tudi) system and kinship-like networks (Kavanagh et al., 2018; Xi, 2024). In this context, social support is a constant structural baseline rather than a fluctuating variable. Since MTM specifically measures “*changes”* in the social environment, it may fail to capture the influence of this enduring, high-level, and unconditional support system. Furthermore, the results suggest a mechanism of internalization. As shown in the model, internal regulatory processes—specifically Emotional Transformation (β = 0.422, *p* < 0.001) and Practice for Change (β = 0.302, *p* < 0.001)—dominated the sustenance phase. This indicates that while the social environment provides the necessary background context, the actual driver for long-term adherence in skill-intensive practices shifts from external social reliance to internal self-regulation and emotional cultivation. This aligns with Self-Determination Theory, suggesting that as practitioners advance, their motivation becomes more autonomous and less dependent on social reinforcement (Teixeira et al., 2012; Yan et al., 2025; Zhu et al., 2025). Taken together, these patterns do not undermine the MTM framework, but instead suggest that its conceptualization of social environment may require contextual refinement for activities in which social influence is embedded within enduring cultural norms and community structures, rather than expressed primarily through discrete interpersonal encouragement. This finding highlights a potentially important contextual distinction in Wushu: enduring relational structures may function more as background conditions for practice, whereas sustained engagement is driven more directly by internalized emotional management and self-regulatory routines.

Theoretically, these findings extend the MTM by providing partial construct-level support while illuminating its context-specific boundaries in a Wushu setting. The strong phase-specific predictors reinforce MTM's dual-structure design, while the non-significant effects of participatory dialogue and changes in the social environment may reflect context-specific characteristics of Wushu practice and the timing at which these constructs were assessed, indicating potential behavioral domains where MTM's constructs may not fully align with contextual realities. Importantly, this misalignment should be interpreted as situational rather than theoretical, suggesting that certain MTM constructs may operate differently across practice stages or populations. This supports calls for expanding behavioral models to incorporate cultural identity, intrinsic motivation, mastery orientation, and embodied learning processes, particularly in culturally meaningful forms of physical activity (Chiva-Bartoll et al., 2021; Wang et al., 2024; Huang and Long, 2025; Prieto-González et al., 2025; Rhodes et al., 2025).

Practically, these findings provide clear direction for promoting Wushu participation. Enhancing the availability, accessibility, and affordability of training environments may substantially increase initiation, as environmental conditions appear to outweigh motivational or evaluative considerations. Instructional strategies that scaffold early skill acquisition and build confidence may further support entry into practice. To sustain engagement, programs should emphasize emotional regulation, goal setting, structured practice routines, and reflective training methods. Given the limited influence of social environment changes, interventions may benefit from fostering deeper community belonging and cultural identification rather than relying solely on peer encouragement or generic social support strategies. These findings also resonate with broader public health perspectives, which emphasize that culturally grounded physical activities yield greater population-level benefits when paired with enabling environments and mechanisms that support long-term participation (Sentell et al., 2024). In the context of Wushu, this implies that expanding structural access for beginners while simultaneously strengthening emotional and self-regulatory capacities for existing practitioners may be particularly effective in promoting sustainable engagement. Such an approach acknowledges both the cultural significance of Wushu and the behavioral processes required for sustained practice, reinforcing its potential as a meaningful component of health promotion initiatives.

Taken together, the present findings clarify the distinct mechanisms that support the initiation and sustenance of Wushu practice and highlight important contextual nuances that shape behavioral engagement in Wushu. By integrating behavioral science with cultural and motivational perspectives, this study advances theoretical understanding of Wushu participation and provides a foundation for future refinement of models that explain culturally embedded physical activities.

## 6 Strengths and limitations

This study offers several notable strengths. It represents one of the first attempts to apply the Multi-Theory Model to both the initiation and sustenance of Wushu practice, thereby extending behavioral theory into a culturally embedded, technically demanding physical activity. A rigorous analytic sequence—EFA, CFA, and SEM—was employed to ensure strong measurement validity and to test theoretically grounded pathways using a sufficiently large and diverse sample. By integrating psychological and contextual predictors, the study provides a comprehensive account of the mechanisms shaping Wushu engagement across different phases of behavior change.

Nevertheless, some limitations warrant consideration. The cross-sectional design restricts causal interpretation and precludes examination of developmental shifts across training stages. Self-reported measures may introduce recall or social desirability bias, though standardized scales and anonymity procedures helped reduce these risks. Although Harman's single-factor test and the poor fit of the one-factor CFA model suggested that common method bias was unlikely to substantially distort the findings, these approaches cannot fully rule out common method variance in self-reported, cross-sectional data. In addition, initiation was assessed retrospectively among individuals who had already engaged in Wushu practice. Accordingly, the initiation-related findings should be interpreted as reflecting perceived factors associated with recalled initiation experiences rather than prospective causal predictors of behavior onset. This design may also be subject to recall bias, survivorship bias, and retrospective cognitive reconstruction. Moreover, the study did not differentiate among Wushu sub-disciplines—such as Taolu, Sanda, and Taijiquan—which may differ in motivational and social dynamics. Finally, although MTM constructs performed adequately psychometrically, the absence of culturally salient variables (e.g., cultural values, identity-related motives, enjoyment) may have constrained the model's explanatory breadth. Furthermore, although the sample was diverse in training experience, learning mode, and competition exposure, the present study did not formally test whether MTM pathways differ across these subgroups. Future research should consider multi-group SEM (e.g., by training experience or competition involvement) or incorporate these characteristics as moderators/covariates to evaluate the stability and boundary conditions of MTM mechanisms in Wushu contexts.

These limitations point to several directions for future research. Longitudinal or intervention-based designs are needed to clarify causal pathways and to observe how MTM constructs evolve over time. Future studies should examine broader and more diverse Wushu populations and compare different Wushu disciplines to assess the stability of the MTM structure. Incorporating cultural identity, intrinsic motivation, and social belonging may enhance the model's applicability to Wushu. Intervention studies targeting environmental access, emotional regulation, and self-regulatory routines would further validate MTM mechanisms and support evidence-informed strategies to promote sustainable Wushu engagement.

## 7 Conclusion

This study extends the Multi-Theory Model into the domain of Wushu by providing partial support for phase-specific MTM patterns related to recalled initiation experiences and current sustenance of Wushu practice. The findings reveal a clear phase-specific pattern: initiation is jointly shaped by changes in the physical environment and behavioral confidence, whereas sustained engagement is more strongly supported by emotional regulation (emotional transformation) and self-directed practice processes (practice for change). Together, these results underscore both the utility of MTM and its contextual boundaries when applied to culturally grounded, skill-intensive activities, highlighting the importance of cultural identity, intrinsic motivation, and community-based structures in shaping long-term participation.

From a practical perspective, the findings suggest that efforts to promote Wushu participation should adopt phase-appropriate strategies. Initiation-oriented approaches may benefit from enhancing environmental accessibility, visibility, and instructional opportunities while simultaneously strengthening behavioral confidence, whereas sustenance-focused interventions should emphasize emotional transformation, habit formation, and reflective practice to support continued engagement. As Wushu continues to evolve within contemporary health promotion and physical activity initiatives, integrating behavioral science with cultural and pedagogical perspectives represents a promising pathway for advancing both research and practice.

## Data Availability

The raw data supporting the conclusions of this article will be made available by the authors, without undue reservation.
